# Combating Dual Challenges in Maize Under High Planting Density: Stem Lodging and Kernel Abortion

**DOI:** 10.3389/fpls.2021.699085

**Published:** 2021-11-02

**Authors:** Adnan Noor Shah, Mohsin Tanveer, Asad Abbas, Mehmet Yildirim, Anis Ali Shah, Muhammad Irfan Ahmad, Zhiwei Wang, Weiwei Sun, Youhong Song

**Affiliations:** ^1^School of Agronomy, Anhui Agricultural University, Hefei, China; ^2^Tasmanian Institute of Agriculture, University of Tasmania, Hobart, TAS, Australia; ^3^School of Horticulture, Anhui Agricultural University, Hefei, China; ^4^Department of Field Crop, Faculty of Agriculture, Dicle University, Diyarbakir, Turkey; ^5^Department of Botany, University of Narowal, Narowal, Pakistan

**Keywords:** kernel abortion, maize, lodging, sugar metabolism, management, stem lodging, field management, grain yield

## Abstract

High plant density is considered a proficient approach to increase maize production in countries with limited agricultural land; however, this creates a high risk of stem lodging and kernel abortion by reducing the ratio of biomass to the development of the stem and ear. Stem lodging and kernel abortion are major constraints in maize yield production for high plant density cropping; therefore, it is very important to overcome stem lodging and kernel abortion in maize. In this review, we discuss various morphophysiological and genetic characteristics of maize that may reduce the risk of stem lodging and kernel abortion, with a focus on carbohydrate metabolism and partitioning in maize. These characteristics illustrate a strong relationship between stem lodging resistance and kernel abortion. Previous studies have focused on targeting lignin and cellulose accumulation to improve lodging resistance. Nonetheless, a critical analysis of the literature showed that considering sugar metabolism and examining its effects on lodging resistance and kernel abortion in maize may provide considerable results to improve maize productivity. A constructive summary of management approaches that could be used to efficiently control the effects of stem lodging and kernel abortion is also included. The preferred management choice is based on the genotype of maize; nevertheless, various genetic and physiological approaches can control stem lodging and kernel abortion. However, plant growth regulators and nutrient application can also help reduce the risk for stem lodging and kernel abortion in maize.

## Introduction

Maize is one of the most widely grown cereal crops and is important for human food, animal feed, industrial raw materials, and biofuel energy (Shiferaw et al., 2011; Anjum et al., 2017). The world population is increasing and is projected to surpass 9.8 billion by 2050 (FAO, 2012). Thus, the crop production must be increased by 50% to the current production level by 2050, to meet food demand of the burgeoning human population (Searchinger et al., 2019; Tanveer, 2020). How to increase maize yield has been a key research question for agronomists for many years (Aslam et al., 2015). The use of high-yield cultivars or hybrid varieties, along with efficient crop husbandry practices, contributes to the increase in maize production; however, there are some inevitable factors, such as climate change, that are still limiting the achievement of maximum yield potential of maize worldwide (Xiao et al., 2008; Martins et al., 2019; Hussen, 2020).

Managing plant density (PD) at the field level is one of the most effective practices and plays a key role in increasing maize yield per unit area (Maddonni and Otegui, 2006; Dhaliwal and Williams, 2020). A low PD results in lower yield production because of smaller number of productive plants per unit area and greater weed infestation (Sharifi et al., 2009; Lashkari et al., 2011; Zhang et al., 2016; Zhao et al., 2019). The use of high PD may provide a high yield (Tokatlidis et al., 2011; Van Ittersum and Cassman, 2013) due to increased leaf area index (LAI) and photosynthetically active radiation (Maddonni and Otegui, 2004; Novacek et al., 2013), and improved dry matter and nitrogen accumulation (Ciampitti and Vyn, 2011; Yan et al., 2017). Conflicting reports have indicated negative effects of high PD, such as mutual shading, intraspecific competition for resources, accelerated leaf senescence, and reduced photosynthesis (Edmeades et al., 2000; Sharifi et al., 2009; Li H. et al., 2010; Li S. K. et al., 2010; Antonietta et al., 2014). In maize, high PD decreased cob length, ear weight, the number of kernels per row, and stalk area by 10.8, 6, 10, and 20% (Testa et al., 2016). Therefore, proper management is required to optimize PD to increase the yield per unit area.

High PD also results in two major problems, stem lodging and kernel abortion, which are casually linked with each other in the context of maize yield. Under intensive crop management, high PD can cause plants to be more susceptible to lodging, which is mainly due to increased stem length and decreased diameter and wall thickness that could diminish flexural rigidity, breaking resistance, and Young's modulus (Wu et al., 2012; Wu and Ma, 2018). High PD increases plant height through greater internode length and smaller stem diameter, promoting the chances of stem lodging further (Wang et al., 2011; Shah et al., 2017). High PD increases competition for nutrients between individual plants, which results in thinner maize stems and a higher risk of lodging (Huang, 2008; Feng et al., 2010; Shah et al., 2017). Moreover, increasing PD also results in reduced quality and intensity of light reaching the maize canopy (Tokatlidis et al., 2011), thus inhibiting the photo-destruction of auxin. More auxin increases the rate of internode elongation, causing the length of internodes to increase (Chen et al., 2018). In addition, plant density influences the quality of light (ratio of red to far-red) during the early growing season and impacts internode length in the lower canopy (Rajcan et al., 2004). Xue et al. (2016a) reported that high PD increases the rate of rapid internode elongation and decreases the duration of rapid internode thickening, causing internodes to increase in length and decrease in diameter. Therefore, as plant density increases, the length of the basal internode significantly increases, whereas the diameter significantly decreases (Novacek et al., 2013). The length of internodes below the ear increases as plant density increases, causing ear and plant height to increase. Elevated ear height increases the center of gravity (Xu et al., 2017). These changes make maize plants more susceptible to bending by the wind. Moreover, under high PD, reduction in mechanical strength and alteration in sugar metabolism also induces stem lodging (Shah et al., 2017; Kamran et al., 2018a).

Kernel abortion is another negative impact of increasing PD in maize, as high PD induces the abortion of young kernels by reducing the ratio of ear growth rate to tassel growth rate (Vega et al., 2001a,b; Sangoi et al., 2002). This reduction favors ear and kernel abortion (Cárcova and Otegui, 2001; Vega et al., 2001a,b). Moreover, high PD increases leaf formation and plant-to-plant competition, accelerates leaf aging, and reduces photosynthesis and the net assimilation rate of individual plants because of decreased carbon and nitrogen supply to the ear, thereby causing kernel abortion (Edmeades et al., 2000; Hiyane et al., 2010; Yan et al., 2010; Antonietta et al., 2014). Under high PD, there is a series of consequences that are detrimental to ear ontogeny and results in barrenness. First, ear differentiation is delayed in relation to tassel differentiation (Sangoi, 2001). Later-initiated ear shoots have a reduced growth rate, resulting in fewer spikelet primordia transforming into functional florets by the time of flowering (Sangoi et al., 2002). Functional florets extrude silks slowly, decreasing the number of fertilized spikelets because of lack of synchrony between anthesis and silking (Sangoi, 2001; Testa et al., 2016). Thus, the risk of kernel abortion increases under high PD because of increased ear height and the development of stems with reduced diameters (Stanger and Lauer, 2007; Novacek et al., 2013). Therefore, kernel abortion and stem lodging in maize under high planting density were discussed in this review.

**Figure 1:**
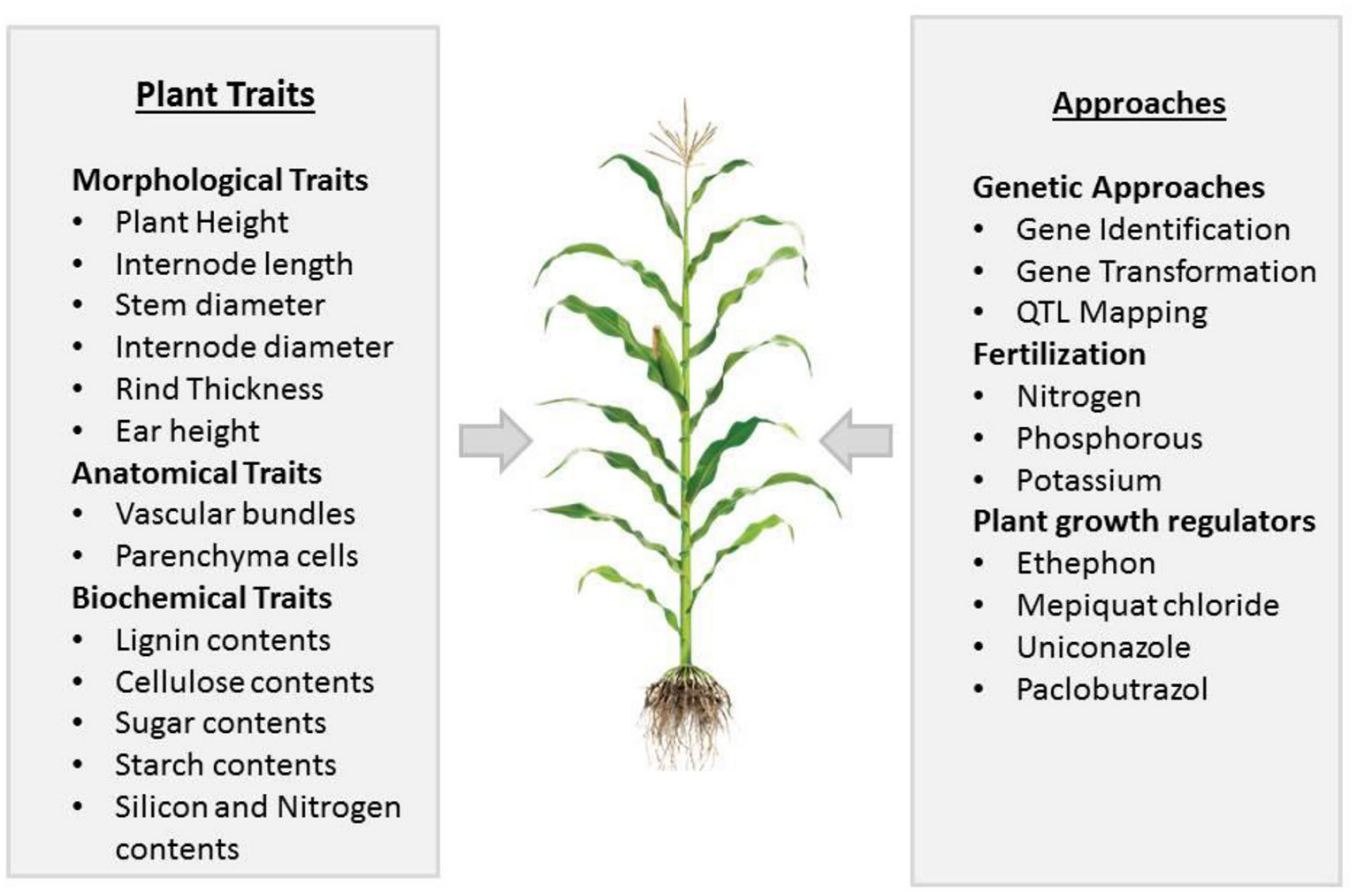
Summary of different plant traits and approaches important in affecting stem lodging and kernel abortion in maize.

Regardless of high PD, stem lodging also causes kernel abortion, and at a high PD, it is very difficult to identify the key player that causes kernel abortion. Some preliminary studies indicated that the first week of pollination is crucial for seed set in maize, and the continuous availability of sucrose for the development of seeds is necessary during and after pollination. If stem bending/breakage occurs in dense maize, which may cause an inadequate supply of sucrose, the abortion of kernels could result, which may range from 20 to 50% (Zinselmeier et al., 1999; McLaughlin and Boyer, 2004; Hiyane et al., 2010). It is noticed that stem lodging in maize effects yields most when it occurs at the reproductive stage (Li et al., 2015), as lodging resulted in stalk breakage, which causes a reduction in nutrient flow for developing grains, inducing kernel abortion (Xue et al., 2020). To improve crop yield and ensure food security, we need to eradicate factors that cause complete crop failure. Along with other factors, kernel abortion is significantly noticed along with stem lodging, making stem lodging a prominent factor that needs to be addressed. This phenomenon has not been discussed before; therefore, in this review, we elucidated the link between stem lodging and kernel abortion in maize under high planting density. This study also reviewed recent knowledge of plant characteristics that are important for improving the resistance to stem lodging and kernel abortion (Figure 1). We also discussed further physiological, genetic, agronomic, and management approaches to reduce maize stem lodging and kernel abortion.

## Grain Yield Loss Caused by Stem Lodging and Kernel Abortion

Stem lodging significantly reduces maize yield by up to 75% (Cheng et al., 2011; Wen et al., 2019); however, yield reduction caused by lodging depends on its timing and the stage of maize growth (Li et al., 2015). Lodging during the 12th leaf and grain-filling stages can reduce maize yield by 30–38 and 45–48%, respectively (Li et al., 2015; Jun et al., 2017). In addition to grain loss, lodging increases harvest cost and reduces grain quality (Huang et al., 2015; Jun et al., 2017; Xue et al., 2018). Lodging-induced yield reduction is significantly associated with reduced carbon assimilation and mineral translocation during the grain-filling stage, and improved respiration and chlorosis, i.e., loss of chlorophyll content and greater vulnerability to pests and diseases (Zhu et al., 2006; Foulkes et al., 2011; Shah et al., 2017). Stem lodging at the reproductive stage is more detrimental than during the vegetative stage because at an early development stage, the logged stem can be re-erected, whereas the stem cannot be re-erected during the anthesis/grain filling stage after lodging, resulting in greater yield reduction (Berry et al., 2000, 2004; Piñera-Chavez et al., 2016; Jun et al., 2017; Shah et al., 2017).

Kernel setting is associated with a source-sink relationship, which is an important determinant of maize yield (Borrás and Otegui, 2001; Borrás et al., 2003; Borrás and Gambín, 2010; Yu et al., 2015). Kernel abortion can account for 8–12% yield loss during the dry season (Cheng and Lur, 1996). During pollination, any biotic or abiotic stress exacerbates the abortion of kernels, which may reduce the number of final kernels and final yield by up to 95% (Rattalino et al., 2011; Novacek et al., 2013; Testa et al., 2016).

## Physiological Regulation of Stem Lodging and Kernel Abortion Under High PD

### Morphophysiological Traits

Plant morphological and physiological traits play important roles in determining stem lodging and kernel abortion under high PD. Stem lodging is one of the most severe constraints on the use of high PD in maize (Argenta et al., 2001).

The major and most important morphological feature associated with stem lodging and kernel abortion under high PD is plant height (Yan et al., 2010; Song et al., 2016; Sher et al., 2018). Tall cultivars may be more susceptible to lodging than shorter plant cultivars, which are more resistant to lodging stress (Li et al., 2011). Decreased plant height results in higher lodging resistance because of lower center of gravity and reduced fresh weight, which minimize the risks of lodging (Ransom, 2005; Echezona, 2007). Additionally, plants with lower height also have a small dry matter, and this decreases grain yield. Therefore, maize cultivars with high resistance to stalk lodging should have a lower ear position to decrease center of gravity, larger leaf spacing, and smaller leaf angle above the ear to allow for more light transport to the mid and lower canopy. This plant type has increased stalk lodging resistance and decreased kernel abortion.

Rind thickness is another morphological feature associated with stem lodging. In various studies, rind thickness, internode diameter, and internode length have been used as predictors of stem strength in sorghum (Teetor et al., 2017). Under high PD, rind strength decreased, as evidenced by a decrease in rind penetrometer resistance, which resulted in smaller diameters and weaker stems that broke easier (Stanger and Lauer, 2007). This was also caused by a decrease in the mechanical tensile strength of maize stems under high PD, which resulted in plant lodging and reduced both the yield and quality of maize (Fu et al., 2013). Additionally, positive correlations have been observed among plant height, stem diameter, internodal length, numbers, and lodging index, and it has been concluded that these traits are substantial plant characteristics that influence the vulnerability of plants to lodging (Table 1).

**Table 1:** Plant traits of maize connected with stem lodging resistance and kernel abortion.

**Traits**	**Crop**	**Connection**	**References**
**Morphological traits**			
Plant height	Maize	Positive	Yokozawa and Hara, 1995; Dong et al., 2006; Wang et al., 2011
Basal internode length	Maize	Positive	Yang et al., 2009; Wang and Frei, 2012; Zhang et al., 2013
Stem diameter	Maize	Positive	Sellmer et al., 2001; Shah et al., 2017
Internodal diameter	Maize	Positive	Zuber et al., 1999; Shah et al., 2017
Rind thickness	Maize	Negative	Zuber and Grogan, 1961; Thompson, 1963; Zuber et al., 1999
**Anatomical traits**			
Vascular bundles	Maize	Positive	Wang et al., 2006
Ear height	Maize	Positive	Stanger and Lauer, 2007; Novacek et al., 2013
Parenchyma cells	Maize	Positive	Dunn and Briggs, 1989; Niklas, 1991; Spatz et al., 1993
**Biochemical traits**			
Lignin contents	Maize	Positive	Zhang et al., 2010; Chen et al., 2011; Loor et al., 2013
Cellulose contents	Maize	Positive	Jones et al., 2001; Tanaka et al., 2003; Shah et al., 2017
Hemicellulose contents	Maize	Positive	Jones et al., 2001; Tanaka et al., 2003; Shah et al., 2017
Sugar contents	Maize	Positive	Setter and Flannigan, 1986; Thomas and Howarth, 2000; Ruan et al., 2012; Shah et al., 2017
Starch contents	Maize	Positive	Hänggi and Fleming, 2001; Loor et al., 2013; Shah et al., 2017
Silicon and nitrogen contents	Maize	Positive	Zhang et al., 2010; Chen et al., 2011

The leaf sheath that surrounds and protects the hollow internodes of a stem also provides plants with great physical support (Hale et al., 2021). In a study, it was found that, on average, the leaf sheath contributed 40, 68, and 38% of the overall stem bending strength, flexural rigidity, and safety factor, respectively, in oat, while it accounted for 11, 24, and 10%, respectively, in wheat plants (Wu and Ma, 2020). Any damage to leaf sheaths may result in the weakening of stem breaking resistance in plants (Wu et al., 2012). Moreover, the leaf sheath of rice varieties showing low stem breaking strength generally died down earlier than varieties having high stem breaking strength (Ookawa and Ishihara, 1992). These studies emphasized the importance of maintaining the vitality of leaf sheath for enhancing stem bending strength.

Leaf angle plays a vital role in the determining the amount of light intercepted by the canopy, and in maize leaves with different leaf angles receive different qualities of light (particularly enriched with far-red, FR) and reduced red (R) radiation under high PD (Lee and Tollenaar, 2007; Hammer et al., 2009). An erect leaf posture improves lodging resistance, and a prostrate leaf stature reduces resistance to lodging under dense planting (Wu and Ma, 2019). A high FR/R ratio triggers many morphophysiological changes in plant architecture, stimulating stem elongation, and favors apical dominance and decrease in stem diameter (Rajcan and Swanton, 2001). Nonetheless, stem lodging and kernel abortion increased, and stem diameter decreased because of mutual shading (Troyer and Rosenbrook, 1983; Valentinuz and Tollenaar, 2004). Such changes make maize stems more susceptible to falls and breakage before kernels reach physiological maturity (Li et al., 2011).

### Anatomical and Biochemical Traits

The anatomical characteristics of plants have a significant effect on lodging and kernel abortion under high PD. Stem lodging occurs under high PD when plant-to-plant competition for light, nutrients, water, and carbohydrates increases between the stem (source) and the ear (sink) within the plant, ultimately reducing the vigor of the sclerenchyma cells around the vascular bundles in the stem (Nielsen, 2006; Stanger and Lauer, 2007).

To reduce lodging potential and ear falls, plants rely on their anatomical features to provide shape and strength, bond cells together, and provide rigidity for the whole plant (Wen et al., 2019). However, the development of anatomical features varies significantly with cells, species, and accessions within species (Brett and Waldron, 1990; Hazen et al., 2003). Pith parenchyma cells play a vital role in stabilizing the stem and reducing the risk of local buckling and collapse (Niklas, 1991; Spatz et al., 1993; Kong et al., 2013). Stem stand ability increases with the thickness of the parenchyma layer because parenchyma cells can absorb the effects of environmental forces, such as light, wind, and rain without heating or mechanical damage (Kokubo et al., 1989). About 50–80% of the strength of a maize stalk comes from its outer structure, the rind (Zuber et al., 1980). Wang T. et al. (2015) reported that the crush strength of maize stalks is significantly positively correlated with the ratio of mechanical tissue thickness to internode radius and the ratio of sclerenchyma thickness to internode radius. Xue et al. (2016a) reported that the number of mechanical cell layers, the thickness of mechanical tissue, and the ratio of cortex thickness to internode radius determine ~79% of the Rind penetration strength (RPS) at the third internode. The mechanical strength of maize stalks depends primarily on the cell wall of mechanical tissues in the internode rind (Leroux, 2012). The main components of the cell wall are structural carbohydrates, such as cellulose, hemicellulose, and lignin (Li S. C. et al., 2003; Li Y. et al., 2003; Wang et al., 2006), and, therefore, stalk strength is significantly and positively related to the contents of these materials (Appenzeller et al., 2004; Chen et al., 2007). Sclerenchyma cells around the vascular bundles in the stem are responsible for mechanical strength, and reduction in sugar supplies can reduce the vigor of sclerenchyma cells, inducing stem lodging (Novacek et al., 2013).

In addition to the anatomical characteristics of maize plants, some basic biochemical properties, such as cellulose, hemicellulose, lignin, and soluble sugar content, are essential and have a significant effect on lodging resistance under high PD. When PD increases, it affects the vigor of sclerenchyma cells around vascular bundles and may reduce the synthesis of total non-structural carbohydrates and proteins, and potassium level in the stem, possibly causing stem lodging by altering the source-sink ratio (Wang et al., 2006). Furthermore, decreased stem protein and sugar levels cause senescence of pith tissue and increase stem lodging (Shah et al., 2017). In another study, Wang and Hu (1991) found that lodging-resistant maize varieties exhibited a higher accumulation of carbohydrates and lignin in the stems than susceptible varieties. A non-significant relationship was also observed between the starch content of the stem and lodging resistance (Zhang et al., 2009). Moreover, the accumulation of cellulose, hemicelluloses, and lignin improved the thickening and flexibility of the culm wall (Jones et al., 2001; Tanaka et al., 2003). Typically, stem strength depends on cellulose and lignin content; therefore, plant stems with lower lignin or cellulose levels are susceptible (Shah et al., 2017).

Lignin is one of the main components of and confers rigidity to cell walls; therefore, it is associated with the mechanical stability of plants (Loor et al., 2013). Low lignin content may result in weak mechanical strength of the cell wall and could easily cause stem lodging. Lignification of the cell wall could improve the stability of the cell wall and increase the physical strength of the stem (Chen et al., 2011). Therefore, lignin is an integral component of plant health and function (Chen et al., 2011). The application of silicon and nitrogen could considerably increase the lignin content in hardened cells and increase cellulose content, thereby reducing stem lodging index (Zhang et al., 2010; Chen et al., 2011).

Multiple reasons are involved in kernel abortion, which may be due to failure in pollination or defective ovary, and even abortion is noticed after successful fertilization (Gustin et al., 2018). Under high PD or under a lodging situation, when sources are deficient, plants are used to abort few of the ovaries as a survival tactic (Ruan, 2014; Tardieu et al., 2014). Reduction in the supply of sucrose and concentrations of assimilates causes kernel abortion (Shen et al., 2018). Many studies have discussed that under scarce sucrose conditions, apical kernels are more likely to abort as they have the weakest sink (Shen et al., 2018).

When it comes to the anatomy of female florescence, the kernel at the base of the ear is less likely to abort because even in the most competitive environment they have more photo assimilate supply as compared with the kernels at the terminal parts, which are more likely to abort. The same is the case under water deficit conditions, increasing PD under water deficit conditions increased the risk of kernel abortion for the kernels at the tip of ears (Setter et al., 2001; Setter and Parra, 2010). It is noted that the expression of the TPP gene (*trehalose phosphate phosphatase*) in the development stage of the ear reduces kernel abortion (Nuccio et al., 2015). Varieties with the TPP transgenes have comparatively lower kernel abortion in high planting density (Hannah et al., 2017). To date, little is known about the molecular mechanism that controls kernel abortion; therefore, there is a need for more studies that are focused on uncovering the exact mechanism of kernel abortion and its reasons. Based on previous studies, there is a hypothesis, on which current studies are relying, that inadequate supplies of carbohydrate and water in the development stage of kernel cause a reduction in kernel set (Gustin et al., 2018) under high PD. The other hypothesis is that reduction in supplies of water causes a reduction in cell expansion, which causes kernels to abort (Oury et al., 2016). The above-mentioned findings have important implications for future maize breeding.

In conclusion, plant characteristics play different roles in controlling the risk of lodging and kernel abortion in maize. These characteristics should be considered in future studies to develop lodging-resistant varieties and reduce kernel abortion.

## Role of Stem Sugars in Lodging Resistance and Kernel Abortion

Stem lodging is a significant constraint to the yield of maize at high PD; even high-yielding hybrids can be affected by it (Betra'n et al., 2003; Flint-Garcia et al., 2003). As stated above, stem lodging significantly depends on the distribution of structural chemical constituents of stems (Appenzeller et al., 2004; Chen et al., 2007). Additionally, the roles of structural and nonstructural carbohydrate and sugar contents in lodging resistance and kernel abortion are largely unknown. In this study, we attempted to summarize how sugar content in shoots relates to lodging resistance and kernel abortion, and the mechanisms involved in sugar biosynthesis, transport, and deposition. Sucrose phosphate synthase (*SPS*) and sucrose synthase genes (*SUS*) play a role in the accumulation of sugars in stems, and effectively minimize stem lodging and kernel abortion (Zinselmeier et al., 1999; Slewinski, 2012; Mizuno et al., 2018).

Two plausible possibilities are given here to elucidate the co-relationship of stem sugars and lodging. The first is that high sucrose content will require more water in parenchyma cells, thereby increasing cell turgor pressure and creating stiffer cells, thereby combating lodging. The second possibility is the presence of higher concentrations of sugar in the vicinity of the cell, which could facilitate carbohydrate mobilization and help the cell to maintain its integrity, which restricts necrosis that degrades dead cells and tissues and, consequently, compromises stem structure. However, sugar accumulation (controlled by *SUS* genes) delays senescence until the end of the season and increases resistance to stem lodging; thus, it minimizes kernel abortion and prolongs grain development (Thomas and Howarth, 2000).

Non-structural carbohydrates play a significant role in stem lodging tolerance and kernel abortion. When photosynthesis is at its peak, non-structural carbohydrates are stored in the parenchyma cells and vascular tissues, adding to the physical strength of the stem, thereby improving plant lodging tolerance. During later stages, when photosynthesis is compromised, stored non-structural carbohydrates act as secondary sources for grain filling, significantly reducing kernel abortion (Slewinski, 2012). Sustaining non-structural carbohydrates in the stem has been proposed as an effective way to control stem lodging (Shiferaw et al., 2011). It can be concluded that cultivars with high stem sugar deposits are more likely to resist lodging.

Kernel abortion is mainly caused by an insufficient carbohydrate supply (source-sink relationship; Shen et al., 2020). In the source-sink relationship, the stem buffer system is mainly dependent on sucrose (Daynard et al., 1969). Sugar transportation is regulated by invertase genes and is always noted from the source to sink organs. Initially, sugar is biosynthesized in the leaf by the action of *SPS* genes used for development, and any surplus is placed in the storage organs by the activity of the *SUS* genes. The stem in the vegetative growth stage acts as the storage organ (sink), but in later stages, during reproductive growth when sugar is needed for kernel development, the sugar content in the stem tissues begins to act as a source, and a flow from stem to kernel occurs (Slewinski, 2012). Therefore, stem sugars can maintain the supply of sugar for kernel development and can play a role as a limiting factor for kernel abortion. However, the role of stem sugars in important metabolic processes has been neglected in the past, and only recently has its roles in lodging resistance, kernel abortion, and other key metabolic processes been addressed (Saini and Westgate, 1999; McLaughlin and Boyer, 2004; Hiyane et al., 2010).

Sucrose in storage organs displays an apoplasmic movement, and its depletion can affect the total sugar content and total stem dry matter (Sayre et al., 1931; Setter and Flannigan, 1986; Slewinski, 2012). Thus, sugar partitioning is also important in relation to lodging resistance and kernel abortion. *ZmSUT1*, a member of the sucrose transporter family, is highly expressed in photosynthetic tissues and is responsible for the mobilization of carbohydrates from source to sink tissues (Carpaneto et al., 2005; Slewinski and Braun, 2010). Braun and Slewinski (2009) suggested that characterization of other family member genes could further clarify the roles of these genes in sugar mobilization, and is discussed in detail in later sections of this article. It will be interesting to discover the involvement of sucrose transporter *SUT* genes in regulating non-structural carbohydrate (NSC) reserves in maize.

**Figure 2:**
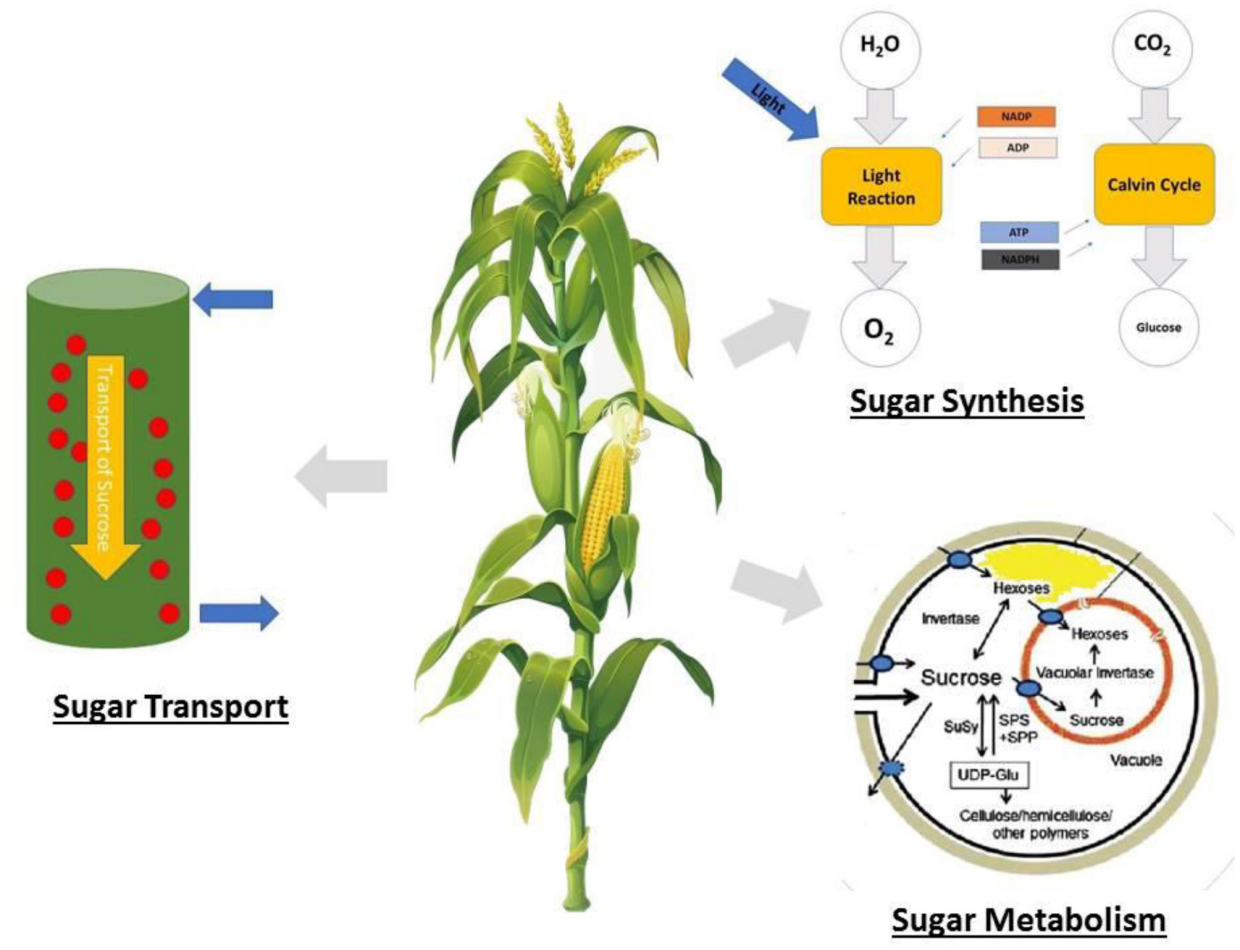
Summary of stem sugar storage and translocation in maize.

In maize, the fertility of the ovaries has a more significant influence on kernel number than that of pollen, which is different from that of wheat, rice, and barley (Boyer and McLaughlin, 2007; Barnabás et al., 2008). During reproductive growth, ovaries require sugar in the form of sucrose for developing embryos, and a reduction in sugar supply results in the abortion of the embryos, resulting in aborted kernels (McLaughlin and Boyer, 2004; Hiyane et al., 2010). Therefore, sugar accumulation should be properly managed to ensure lodging resistance and minimal kernel abortion, because all these factors have an impact on the final yield. This must be understood for an in-depth elucidation of sugar metabolism and transport. A summary of stem sugar storage and translocation in overcoming stem lodging and kernel abortion is shown in Figure 2.

### Sugar Biosynthesis and Metabolism

**Figure 3:**
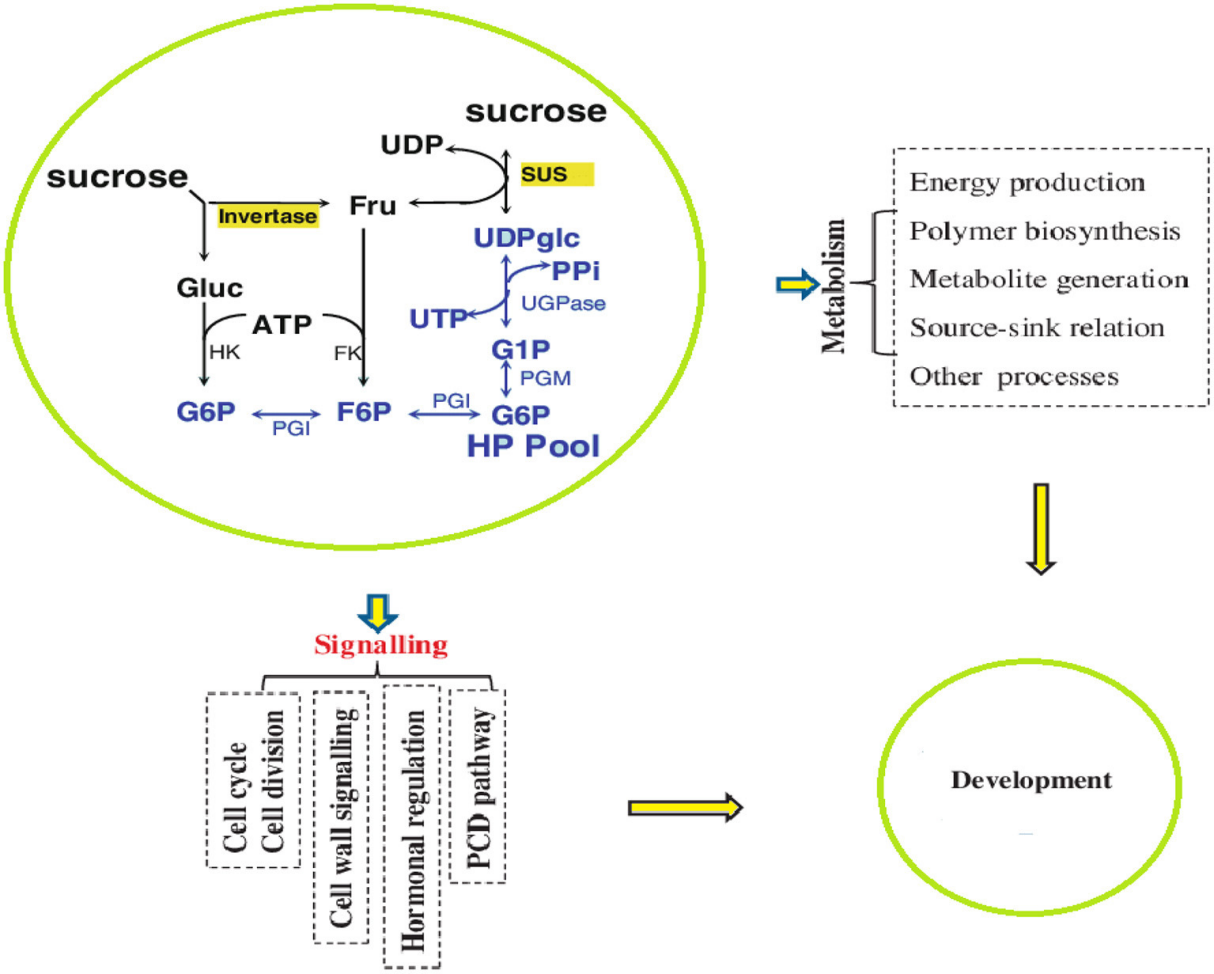
Summary of sugar biosynthesis and metabolism.

Sugar biosynthesis and metabolism are complex processes affected by different growth and environmental conditions. Briefly, as a result of photosynthesis, sugar is synthesized in the form of sucrose by the catalytic activity of sucrose-phosphate synthase (SPS) and converted into ADP-glucose or UDP-glucose, which is used in plant metabolism by the action of the SuSy enzyme (Hendriks et al., 2003; Kolbe et al., 2005). The SuSy enzyme is encoded by *SUS* genes that may be present in the cytoplasm, cell wall, and vacuoles (Stein and Granot, 2019). Plants with low activity of *SUS* genes exhibited stunted growth, whereas the overexpression of *SUS* genes produced significantly increased growth and strong cell structure with thickened cell walls (Stein and Granot, 2019; Figure 3). At the cellular level, the intercellular biosynthesis of sugar and starch is initiated by ADP-glucose pyrophosphosphorylase (AGPase), which provides ADP-glucose (Geigenberger, 2011). AGPases are primarily encoded in the cytosol and are minimally encoded in amyloplasts and plastids (Burton et al., 2002).

After biosynthesis, sugars reach the sink tissues *via* the phloem tubes either through the symplast or apoplast pathway. In the apoplasmic unloading pathway, sucrose is unloaded into the cell wall matrix from the sieve element/companion cell (SE/CC) complex mediated by sugars will eventually be exported transporter (SWEET) proteins, which are typically localized on the plasma membrane, to transport sucrose or hexoses down a concentration gradient in an energy-independent manner (Shen et al., 2019). Within the cell wall, sucrose is often hydrolyzed by cell wall invertase (CWIN; with an optimum pH of 5–6) into glucose and fructose (Ruan et al., 2010). Hexoses are then taken up by H^+^-coupled hexose transporters (HXTs; Ruan, 2014). It is common for CWIN and HXTs to be co-expressed in the apoplasmic unloading region, indicating a synergistic functional relationship between CWIN and HXTs (Weber et al., 2005; Jin et al., 2009; McCurdy et al., 2010; Shen et al., 2019). The direct transfer of sucrose can also occur through plasmodesmata, where vacuolar invertase genes hydrolyze sucrose. SuSy and invertases cleave sucrose differently. SuSy proteins cleave sucrose into reversible UDP-glucose and ADP-glucose, whereas cell wall and vacuolar invertases catalyze an irreversible conversion of sucrose into glucose and fructose (Ma et al., 2019).

Additional sugars are transported to the sink tissues, which are the stem and roots in the case of maize, to be stored and added to the organic matter structural carbohydrates (Ruan, 2014; Stein and Granot, 2019). In maize, the involvement of *SUS* genes in starch synthesis was first studied by Chourey and Nelson (1976) who found that gene mutants exhibited a 90% reduction in SuSy and an overall 40% reduction in starch accumulation. However, in mutants with overexpressed *SUS* genes, a considerable increase in the accumulation of ADP-glucose was observed (Li et al., 2013). *SUS* genes appear to play a role in the development of young photosynthetic tissues and starch accumulation in the non-photosynthetic tissues of the plant (Hänggi and Fleming, 2001). Additionally, *SUS* genes were localized in the xylem tissue, and their overexpression resulted in increase in xylem cell wall thickness (Coleman et al., 2006; Bahaji et al., 2014; Wei et al., 2015). Hence, SuSy is responsible for directing carbon to cellulose synthesis, which is used in the development of xylem tissues (Barratt et al., 2009).

This raises the question regarding whether these genes protect plants from lodging and kernel abortion. Four cell wall invertase genes, two vacuolar invertase genes, and 21 invertase isogenes are identified in maize (Juarez-Colunga et al., 2018). Grain yield relies mainly on grain number, size, and starch content (Ngoune Tandzi and Mutengwa, 2020). Maize mutants with higher expression of cell wall invertase resulted in a 1.5-fold increase in production, a 20% increase in starch content, and a noticeable increase in the size of ears, grain size, and numbers, which relates invertase genes with resistance to lodging and kernel abortion. Higher activity of cell wall invertase results in increased shoot strength and sugar content, along with a significant increase in seed number and size (Li et al., 2013). It has also been documented that invertases regulate the production of other plant hormones, such as indole acetic acid (IAA), which directly affects kernel development (Chourey et al., 2010). The expression of genes involved in starch biosynthesis and accumulation is controlled by the number of transcription factors; *ZmNAC128* and *ZmNAC130* transcription factors are involved in sugar accumulation and *ZmEREB156* is involved in the regulation of key genes involved in sugar biosynthesis (Huang et al., 2016; Zhang et al., 2019). From these studies, it is inferred that invertase genes can improve lodging resistance in plants.

### Genes Involved in Sugar Transport

Photosynthetic tissues are the powerhouses of plant sugars that are biosynthesized into different forms. Later, they are translocated to other organs, accumulated, and used in different development and reproduction processes. Processes involved in the translocation have been well-discussed, but little is known about the genes involved in sugar translocation and accumulation. The carbohydrate partitioning defective1 (*Cpd1*) gene plays a role in the early production of phloem and controls the distribution of carbohydrates throughout the plant (Julius et al., 2018). In the case of low expression of *cpd1*, plants are unable to transport sugars from the source to sink tissues, which results in the deposition of sugars in leaves and reduction in sink tissues (stems and roots) causing stunted growth, decreased sturdiness of the stem, and delayed silking and anthesis, which affect kernel size and weight (Julius et al., 2018). Agpsemzm and Agpllzm enzymes play roles in the regulation of starch levels in different tissues (Julius et al., 2018).

In addition to the phloem sieve tissues, other genetic factors are involved in the adequate distribution of carbohydrates throughout plants. Initially, sugars are uploaded to cell walls by the SWEET efflux proteins from source tissue cells, and then to sieve elements and companion cell complexes by sugar transporters (Julius et al., 2017). Sucrose moves passively from high-concentration tissues to low-concentration tissues; sucrose transferase (*Sut*) genes are responsible for the regulation of this symplasmic and apoplasmic movement of sucrose (Aoki et al., 1999, 2003; Zhang C. et al., 2014). Maize has apoplasmic sucrose movement null mutants for the *ZmSut1* gene, which identifies its function as a phloem loading with sucrose from source cell apoplastic regions. Retardation of the *ZmSut1* gene results in slow growth and deposition of sucrose in maize leaves (Slewinski et al., 2009; Slewinski and Braun, 2010).

For the identification of *ZmSut2* gene functions, null mutants were analyzed, and showed a reduction in growth and decreased ear length and kernel number, indicating that *ZmSut2* is responsible for resistance to kernel abortion and contributes to maintaining yields under high-density planting (Leach et al., 2017). Sucrose transporter genes also directly influence maize growth (Leach et al., 2017). Along with the *ZmSut* genes, *ZmSWEET13s* (a, b, and c) genes are involved in the translocation from the sink to source tissues, and knockout mutants showed low expression of genes involved in photosynthesis and carbohydrate metabolism (Bezrutczyk et al., 2018).

As the kernel is the main determinant of crop yield, the genetic factors involved in the transportation of sugars and their accumulation need to be elucidated. *ZmSWEET4c* has been identified in maize and is responsible for the transport and deposition of sugars across the basal endosperm transfer layer, which controls nutrient flow in and out of seeds (Sosso et al., 2015). In wild-type maize, the expression of *ZmSWEET4c* increases four times that of normal, along with the flow of maximum sugar to developing seeds, thereby supporting seed setting (Chourey et al., 2012). Along with *ZmSWEET4c*, another gene involved in sugar transport is *ZmMn1*. The expression of these genes induces the expression of *ZmMRP1*, which prepares the cell machinery for sugar transport (Sosso et al., 2015).

### Linking Stem Lodging With Kernel Abortion Under High PD in Maize

A high PD can increase biomass and yield, but it also increases competition for nutrients and light interception among individual plants (Craine and Dybzinski, 2013). Moreover, plant morphogenesis, stem carbohydrate accumulation, cortex tissue, and mechanical strength are affected by high PD. Thus, maize plants under high PD are more vulnerable to stem lodging (Xue et al., 2016a; Jun et al., 2017). High PD promoted the abortion of kernels before the beginning of grain filling in maize (Sangoi, 2001; Ruffo et al., 2015). Abortion causes permanent losses in seed production and may result in decreased productivity (Salter and Goode, 1967; Hiyane et al., 2010). Floral abortion in maize disrupts carpel development (Hiyane et al., 2010). During the flowering stage, the first week of pollination is crucial for the seed set, during and after pollination. The continuous availability of sucrose for the development of seeds is necessary where an inadequate supply of sucrose results in the abortion of kernels in maize (McLaughlin and Boyer, 2004). Kernel abortion is associated with inhibited photosynthesis and reduced sugar supply to the stems under shade (McLaughlin and Boyer, 2004).

Additionally, under high PD, less interception of light decreases the synthesis of cellulose and lignin in maize, which may cause lodging (Li et al., 2008; Xue et al., 2016b). Stem lodging reduces photosynthesis (Setter et al., 1997; Ma et al., 2014) because of reduced photosynthetic activity, which ultimately reduces the sugar flow in plant storage tissues for metabolic processes. This results in a reduced supply of sucrose to developing kernels, which induces abortion (McLaughlin and Boyer, 2004; Hiyane et al., 2010). However, attack by corn borers increases the occurrence of lodging, reducing CO_2_ assimilation, biomass accumulation, and carbohydrate partitioning, resulting in yield reduction from water disruption and nutrient translocation to the ear, and pre-harvest losses because of stem lodging and dropped ears (Riedell and Reese, 1999; Steffey and Gray, 2002; Rice, 2006).

Under high PD or lodging situations, reduction in the supply of sucrose and concentrations of assimilates causes kernel abortion (Shen et al., 2018). Many studies have discussed that under scarce sucrose conditions, apical kernels are more likely to abort, as they have the weakest sink (Shen et al., 2018). Kernel abortion can be minimized by adequate supplies of N, organic, and inorganic substances, and improving carbohydrate availability during the pollination stage (Paponov et al., 2020). At high PD, many kernels may not develop an event that occurs in some hybrids following poor pollination resulting from a silking period that is delayed relative to tassel emergence (Otegui, 1997) and/or owing to a limitation in assimilate supply that causes the grain and cob abortion in maize (1985). As stated above, there is no direct relationship between high PD and stem lodging and kernel abortion is mentioned in the literature. However, we attempted to draw a causal link between stem lodging and kernel abortion under high PD. This link needs to be validated in future studies.

## Management of Stem Lodging and Kernel Abortion in Maize

### The Physiological and Genetic Approach

#### Genes Involved in Lodging Resistance and Kernel Abortion Reduction

Several studies have shown that various genes are involved in lodging resistance and kernel abortion reduction (Table 2). In cereals, stem diameter, rind penetrometer resistance, and stalk bending strength are determinants of stem lodging (Shah et al., 2017). In more than 250 inbred lines, a genome-wide association study was designed to identify the nucleotides involved in these quantitative traits. A total of 423 QTNs was identified in maize, in addition to 63 cyclin-dependent kinase coding and steroid-binding genes that were identified. Seven of these were classified as transcription factors. These genes are likely involved in cell elongation, stem girth improvement, and development, hence, adding up resistance to lodging; 17 genes for stem stalk bending, 19 for strength/diameter, and 30 for rind penetrometer resistance were identified (Hu et al., 2013; Dante et al., 2014; Zhang et al., 2018).

**Table 2:** Genes involved in lodging resistance and kernel abortion reduction.

**S. No**.	**Genes name**	**Chromosomes numbers**	**Functions**	**References**
1	GRMZM2G119357	1	Chromatin remodeling protein EBS	Pineiro et al., 2003
2	GRMZM5G856734	1	Membrane steroid-binding protein 1	Yang et al., 2008
3	GRMZM5G855808	2	Tetratricopeptide repeat (TPR)-like superfamily protein	Munoz-Martinez et al., 2012
4	GRMZM2G156016	3	Transcription factor VOZ1	Kumar et al., 2018
5	GRMZM2G093276	7	ZIP zinc/iron transport family protein	Fu et al., 2017
6	GRMZM2G073934	5	Tetratricopeptide repeat (TPR)-like superfamily protein	Munoz-Martinez et al., 2012
7	GRMZM2G155312	1	Leucine-rich repeat protein kinase family protein	Imkampe et al., 2017
8	GRMZM2G442523	2	Sugar transport protein 5	Han et al., 2017
9	GRMZM2G029692	7	Protein kinase superfamily protein	Lehti-Shiu and Shiu, 2012
10	GRMZM2G082586	7	DNA binding protein bHLH-transcription factor 105	Zheng et al., 2019
11	GRMZM2G156692	7	proline-rich family protein	Wong et al., 2019
12	GRMZM2G375975	8	Putative MAP kinase family protein	Kong et al., 2013
13	GRMZM2G324276	1	Acetylglucosaminyltransferase family protein	Guelette et al., 2012
14	GRMZM2G083504	2	Transcription factor bHLH62	Lehti-Shiu and Shiu, 2012
15	GRMZM2G160400	7	Spermidine hydroxycinnamoyl transferase	Peng et al., 2019

Other than these factors, lignin is also an evident factor that determines resistance to lodging (Frei, 2013). The maize miR528 family (miR528a and miR528b) regulates the expression of lignin synthesis genes *ZmLAC3* and *ZmLAC5* in an abundant nitrogen environment, and these genes are highly expressed in the internodes and are responsible for lignin deposition in the maize stem (Sun et al., 2018). Knocking down miR528 results in higher expression of ZmLAC3 and LAC5, which results in higher concentrations of lignin more resistant to lodging (Sun et al., 2018). Somssich (2020) identified the LAC10, PRX42, PRX72, PRX52, and PRX71 proteins as responsible for lignification in stems.

### QTLs Involved in Lodging and Kernel Abortion Resistance

Marker-assisted quantitative trait loci (QTLs) are one of the approaches that can be used for the identification of genes and to interrelate qualitative, quantitative, chemical, physiological, and morphological traits. In maize, 22 QTLs for lignin, 2 for starch content, 7 for hemicellulose, and 11 for cellulose were identified, which increased lodging resistance and reduced kernel abortion (Santiago et al., 2016). One QTL for stem sugars was identified to match the *SWEET4-3* putative gene locus in maize (Mizuno et al., 2016). Under high PD, plants encounter 43 different QTLs for six different traits: plant height, ear height, reduced stem diameter, delayed days to tassel, delayed days to silk, and anthesis-silking interval (Yuan et al., 2012).

Different QTLs can be identified using a marker-assisted approach. QTLs for lodging resistance mostly overlap with QTLs for stem strength, which reveals factors responsible for stem strength directly related to lodging resistance (Table 3). QTLs were found on chromosomes 2, 4–8, 9, and 10, which are accountable for root lodging. Additionally, 28 QTLs were found in maize with respect to kernel size and shape (Farkhari et al., 2013). Considering QTLs, their heritability, and other genetic factors responsible for quantitative traits and resistance to lodging can be useful for transforming the food chain and ensuring food security.

**Table 3:** Number of quantitative trait loci identified for stem lodging resistance and kernel abortion reduction in maize.

**Characteristics names**	**Quantitative trait locus numbers**	**Affects**	**References**
Lignin contents	22	Resistance to lodging and kernel abortion	Santiago et al., 2016
Starch contents	2	Resistance to lodging and kernel abortion	Santiago et al., 2016
Cellulose	11	Resistance to lodging and kernel abortion	Santiago et al., 2016
Hemicellulose	7	Resistance to lodging and kernel abortion	Santiago et al., 2016

### Agronomic Approaches

#### Planting Time and Method

Planting time influences lodging intensity and kernel abortion in maize (Angel et al., 2019; Zhang et al., 2019). Delays in planting time accelerates plant growth between seedling emergence and silking, which minimizes crop exposure to cumulative incident radiation during the vegetative process. Therefore, lodging may be reduced if crops are sown at the optimum time (Dahiya et al., 2018). However, the optimum planting time differs with the area and environmental conditions (Andrade et al., 1996; Bruns and Abbas, 2006). Similarly, planting time affects kernel abortion (Zhang et al., 2019), and both early and late plantings can cause kernel abortion and reduce the cumulative intercepted photosynthetically active radiation because of delayed leaf area development and high temperature (Otegui et al., 1995; Zhang et al., 2019). During early planting, the effective grain-filling duration is shortened, owing to the maximum daily high temperature from the silking to the blister stage. Similarly, the grain-filling of apical kernels is restrained, which leads to an increase in kernel abortion (Zhang et al., 2019). Contrarily, delayed planting resulted in significant reductions in final kernel number per unit area, and the number of ears at harvest (Cirilo and Andrade, 1994). Moreover, it was suggested that with delayed planting date, radiation and thermal time during the grain-filling period decreased, leading finally to decreased maize kernel weight (Cirilo and Andrade, 1994; Zhou et al., 2017). Li Y. et al. (2003) reported that with delay in sowing date at Shihezi, Xinjiang Uygur Autonomous region, China, grain-filling rate and final kernel weight decreased. Kernel abortion under late planting time is highly associated with less pollen availability, delayed anthesis and silking of individual plants, anthesis-silking interval, decreased number of exposed silks per apical ear, number of pollen grains per square meter, and kernel number per ear (Uribelarrea et al., 2002). Therefore, optimum planting time can improve maize productivity under relatively appropriate climate conditions and with the avoidance of abiotic stresses during the critical period of kernel formation and growth (Arnold and Monteith, 1974; McMaster and Wilhelm, 1997).

In addition to planting time, planting methods also influence the susceptibility of maize plants to lodge and kernel abort (Bakht et al., 2011; Deng et al., 2019). Different planting methods are practiced worldwide at the time of sowing maize crops (Arif et al., 2001; Bakht et al., 2011; Deng et al., 2019). Inappropriate planting methods can result in sterile plants. Ears and plant size remain small, and crops become susceptible to lodging, diseases, and pests, resulting in lower yield per unit area (Liu and Yong, 2008; Bakht et al., 2011). Abdullah et al. (2008) reported that the ridge planting method was better compared with other planting methods examined (bed and flat methods). The ridge planting method reduces lodging and provides good soil conditions for root development and efficient use of irrigation water and nutrients for proper development (Bakht et al., 2006, 2011; Liu and Yong, 2008). Heat stress during fertilization and during flowering significantly reduces pollen viability and seed yield (Edreira et al., 2011; Wu et al., 2020), while adopting an appropriate planting method may alleviate such high temperature-induced negative effect on pollen development. Tao et al. (2013) reported that the ridge planting method enhances the ability of maize to resist heat stress during the grain filling stage, which may reduce kernel abortion. Likewise, the adoption of ridge-furrow with plastic film mulching resulted in reduced lodging index as compared with the well-watered planting method (Li et al., 2020; Li and Li, 2021).

#### Fertilization

Increasing the planting density and applying N fertilizer are very effective agronomic strategies for raising the yield of modern maize cultivars, but high plant density and excessive N application have led to thinner and taller stalks and increased lodging risk (Li H. et al., 2010; Ciampitti and Vyn, 2012; Shah et al., 2017). Higher nitrogen doses can increase the elongation rate and length of the basal internode and significantly reduce the cellulose content of maize stems (Rajkumara, 2008), thereby decreasing stem strength and increasing lodging rate. Wei et al. (2008) stated that N also increases the development of the upper plant canopy, which decreases basal internode length and, consequently, increases stem lodging. Because of dense canopy development, low light conditions occur, which tend to cause plants to grow vertically, resulting in the development of long internodes and stems with small diameters, and less lignification. This can be managed by the split application of N, rather than high concentration. N application timing should also be considered. During reproductive stages, N application is discouraged to avoid kernel abortion (Bian et al., 2017). Wang et al. (2020) reported that nitrogen application in splits can improve stem lodging resistance of maize under high PD.

Lack of kernel development and enhanced abortion is caused by insufficient supply of carbon and nitrogen assimilates in the ear (Hammad et al., 2020). Nitrogen deficiency may cause kernel abortion, resulting in infertility (Marahatta, 2020). Plants grown under high N levels and high PD have decreased kernel numbers because the floret set is established before silking (Gonzalez et al., 2019; Mueller et al., 2019; Paponov et al., 2020). Thus, an adequate supply of N during the lag phase of the grain-filling stage may reduce kernel abortion (Below et al., 2000; Mueller et al., 2019; Paponov et al., 2020). N deficiency decreases the number of kernels per cob by decreasing maize kernel growth and development (Savin et al., 2006). Optimum N fertilization may increase kernel quantity and weight, resulting in higher crop growth rates, while N deficiency decreases photosynthate production in plants (Worku et al., 2012), consequently reducing the kernel-filling period. Nitrogen deficiency in maize during vegetative growth can cause early maturity (Sharifi and Namvar, 2016) and consequently reduce the kernel-filling period (Mayer et al., 2012). Increasing N availability stimulated ear growth during the bracketing-silking period and during the fast grain-filling phase, consequently resulting in greater maize grain yield (Ning et al., 2018). Moreover, in developing ears, N fertilization likely enhanced the cleavage of sucrose to glucose and fructose in the cob prior to and at silking and the synthesis from glucose and fructose to sucrose in the kernels after silking, thus increasing kernel setting and filling (Ning et al., 2018). Therefore, proper N fertilization can assist in managing kernel abortion and stem lodging in maize under high PD. However, more focus is required to study N application timing and rate, as higher N application is also not beneficial under high PD.

The role of potassium (K) is less evident; but to some extent, it contributes to lodging resistance. An inadequacy of K leads to reduced culm length, diameter, and wall thickness, and plants with inadequate K fertilization exhibit weaker culms than those with proper K fertilization (Mulder, 1954; Shah et al., 2017). K plays an important role in the physical strength of the plant and considerably reduces the lodging index and kernel abortion (Zhang et al., 2010). K can promote lignification in thick-walled cells, thicken collenchyma cells, and increase cellulose content, which reduces the lodging index (Shah et al., 2017). In other studies, the application of K during early stalk development and flowering stages increased structural carbohydrate accumulation and stem strength (Liebhardt and Murdock, 1965; Sun et al., 1989; Li et al., 2012). K deficiency in plants often results in the accumulation of sucrose in source leaves because of insufficient loading of the phloem (Zhao et al., 2001; Cakmak, 2005; Shahzad et al., 2017). K is the major cation in the phloem, and deficiency can lead to poor functioning, including phloem-disrupted metabolism and transport of assimilates, which induces kernel abortion (Shahzad et al., 2017). Epron et al. (2016) and Shahzad et al. (2017) reported that K foliar application may reduce kernel abortion, probably by affecting the phloem transport of assimilates. In another study, it was found that K application increased fertilization by adjusting the period between tasseling and silking, which resulted in a greater number of grain rows, grain cob^−1^, and produced higher grain weight cob^−1^ (Ur Rehman and Ishaque, 2011). Moreover, K, in combination with N, has a synergistic influence on the uptake, translocation, and utilization of nutrients, and it reduces the percentage of senescent stalks, lodging and increases crushing strength and rind thickness (Bukhsh et al., 2012).

#### Growth Regulator Application

Plant growth regulators are artificial chemical compounds used to decrease plant height and other lodging-related plant traits. Some regulators have recently been introduced to control maize lodging (Schluttenhofer et al., 2011). Plant growth regulators can optimize plant morphology and increase yield by regulating endogenous plant hormone signaling and metabolism (Naeem et al., 2012; Zeng et al., 2012). Different plant growth regulators have been applied to maize, such as ethephon (Shekoofa and Emam, 2008), mepiquat chloride (Kamran et al., 2018c), paclobutrazol (Kamran et al., 2020), and uniconazole (Schluttenhofer et al., 2011), in the context of lodging resistance and maize yield improvement; however, the effect on vegetative (and perhaps generative) plant growth is highly dependent on the time of application and dosage of plant growth regulator and probably varies with the maize cultivar used (Hütsch and Schubert, 2018).

Ethephon (2-chloroethyl phosphonic acid) is a plant growth regulator that inhibits stem elongation and promotes stem thickness, thereby improving plant morphological resistance to lodging (Li et al., 2019). Shekoofa and Emam (2008) reported that the application of ethephon is associated with reductions in plant height, leaf area index, and crop growth rate, decreasing lodging by 85–93%, resulting in better kernel filling but also slightly decreasing yield by 2–6% (Khosravi and Anderson, 1991). Yield reduction in maize increases with the application rate of ethephon (Earley and Slife, 1969). However, in another study, ethephon with diethyl aminoethyl hexanoate (DA-6) could offset the yield. Furthermore, the combination of ethephon and DA-6 shortened the length and increased the diameter of internodes below the ear position, improving lodging resistance and the yield of maize (Dong et al., 2006). Ethephon has been observed as a negative regulator of kernel development or grain yield maize because of its negative effects on leaf area development, crop growth rate, and photoassimilate reduction (Shekoofa and Emam, 2008; Gao et al., 2009). However, it has been shown that the application of low concentration of ethephon increases maize kernel yield under high PD (Gao et al., 2009). Given that, more physiological studies are required to explore the role of ethephon further in managing stem lodging and kernel abortion at the same time.

Mepiquat chloride is also a plant growth regulator that mainly reduces the length of internodes in dense plant populations and increases resistance to lodging (Kuai et al., 2015; Kamran et al., 2018b). Kamran et al. (2018b) stated that mepiquat chloride in dense maize populations reduces plant height and ear height and increases stem diameter and lignin content in basal nodes, which ultimately reduces kernel abortion and enhances lodging resistance. The ability of mepiquat chloride to reduce the percentage of lodging results in a more uniform canopy, which further improves grain yield at high density (Kamran et al., 2018b). Similarly, Zhang Q. et al. (2014); Zhang et al. (2017) reported that the application of growth regulators significantly increased the number of kernels per ear and maize yield by increasing the optimal plant density by reducing the lodging percentage.

Paclobutrazol is also a plant growth regulator that mainly reduces the length of the second internode, resulting in reduction in plant height and increased resistance to lodging (Peng et al., 2014). Furthermore, stem diameter, lodging resistance, lignin accumulation, and antioxidant activities were positively affected by its use. Additionally, several studies (Özmen et al., 2003; Dong et al., 2006; Cai et al., 2014) have shown that the canopy of plants is best established by its use, with a significant increase in photosynthetic activity and yield (Wang C. et al., 2015; Xu et al., 2017). Thus, the use of these plant growth regulators increases plant resistance to lodging (Peng et al., 2014; Wang C. et al., 2015).

The application of paclobutrazol increased the mechanical strength of basal internodes and decreased internode length and plant and ear height, thereby ultimately reducing lodging in maize (Kamran et al., 2018a). They also showed that paclobutrazol treatments significantly (*P* < 0.05) enhanced the ear characteristics (ear length and diameter, kernels ear^−1^, and 1,000 kernel weight) and grain yield of summer maize when compared with control treatments (Kamran et al., 2018a). The increase in grain yield, in response to paclobutrazol, is attributed partly to decreased investment in above-ground parts, due to a relatively stouter canopy of paclobutrazol-treated plants, as well as enhanced grain filling, in the treated plants due to improved rooting system, which possibly increased the uptake of nutrients and water (Qi, 2012; Kamran et al., 2018b). Given the above findings, at anthesis, the start and duration of pollen production, the start of silking, and anthesis-silking interval were mostly unaffected by paclobutrazol application (Hütsch and Schubert, 2021). Nonetheless, maize grain yield improvements after paclobutrazol application have been attributed to better grain-filling due to broader canopy, delayed onset of senescence and, thus, the start of chlorophyll degradation and improved rooting system (Kamran et al., 2018b, 2020). Kamran et al. (2020) pointed out that higher photosynthetic rate and duration (longer duration of green leaf area) were thought to be mainly responsible for grain yield increases after paclobutrazol treatment.

Uniconazole, a plant growth regulator used mainly to retard plant growth, results in shorter internodes, thereby increasing stem diameter, strengthening the overall stem structure, and increasing lodging resistance (Sellmer et al., 2001). Particularly in maize, the use of uniconazole results in decrease in plant height because of decrease in gibberellins, which results in reduction in cell length but not a reduction in the number of nodes (Schluttenhofer et al., 2011). However, in buckwheat, the results are not much different; lodging resistance and the lodging index were significantly reduced because of reduced plant height and increased lignin content (Wang C. et al., 2015). Schluttenhofer et al. (2011) reported that uniconazole increases lignin content, mechanical strength of the culm, and rind penetration strength, and decreases plant and ear height, which reduces the risk of lodging stress in maize. The application of uniconazole improved maize grain yield by higher kernel number per cob and increased kernel weight due to enhanced seed filling (Ahmad et al., 2018a,b, 2019). A significant increase in cob size was observed when uniconazole was applied at early growth stages, pointing to the impact of application time on maize yield performance (Xu et al., 2004); thus, considering the time of application may result in increased stem lodging and reduced kernel abortion simultaneously under high PD.

## Conclusion

Stem lodging and kernel abortion considerably reduce grain yield. This review provides an understanding of stem lodging and kernel abortion mechanisms in maize. Interestingly, we found that genes involved in starch biosynthesis and transportation metabolism are involved in stem lodging resistance and kernel abortion. However, targeting sugar metabolism and *via* agronomic management, stem lodging and kernel abortion can be reduced under high planting density. Although there is no direct relationship between all the discussed agronomic practices and kernel abortion; however, reducing stem lodging can reduce kernel abortion in maize under high planting density. Thus, further research should be designed to investigate the genes directly involved in stem lodging and, thereby, induce kernel abortion. This will improve our understanding of the molecular basis of maize resistance to lodging. More focused research is needed to elucidate how sugar synthesis, transport, and accumulation processes should be altered to maximize maize resistance to stressful stimuli under high planting density. Further research should be designed to investigate the genes directly involved in stem lodging, and, thereby, induce kernel abortion. This will improve our understanding of the molecular basis of maize resistance to lodging.

## Author Contributions

ANS, MT, AA, and YS conceived this review and drafted and finalized the paper. YS and MY helped to improve the draft by providing useful suggestions and information. AAS, MIA, ZW, and WS helped in finalizing the paper. All authors approved the work for publication.

## Funding

The work was supported by a grant from China National Key R&D Program (Nos. 2017YFD0301307 and 2017YFD0300204-3) and Anhui Agricultural University Postdoctoral fellowship for ANS.

## Conflict of Interest

The authors declare that the research was conducted in the absence of any commercial or financial relationships that could be construed as a potential conflict of interest.

## Publisher's Note

All claims expressed in this article are solely those of the authors and do not necessarily represent those of their affiliated organizations, or those of the publisher, the editors and the reviewers. Any product that may be evaluated in this article, or claim that may be made by its manufacturer, is not guaranteed or endorsed by the publisher.
